# Adverse Selection? A Multi-Dimensional Profile of People Dispensed Opioid Analgesics for Persistent Non-Cancer Pain

**DOI:** 10.1371/journal.pone.0080095

**Published:** 2013-12-02

**Authors:** Kris D. Rogers, Anna Kemp, Andrew J. McLachlan, Fiona Blyth

**Affiliations:** 1 The Sax Institute, Sydney, New South Wales, Australia; 2 Sydney Medical School, University of Sydney, Sydney, New South Wales, Australia; 3 School of Population Health, The University of Western Australia, Perth, Western Australia, Australia; 4 Faculty of Pharmacy, University of Sydney, Sydney, New South Wales, Australia; 5 Centre for Education and Research on Ageing, Concord Hospital, Sydney, New South Wales, Australia; University of Washington, United States of America

## Abstract

**Objectives:**

This study investigates utilisation patterns for prescription opioid analgesics in the Australian community and how these are associated with a framework of individual-level factors related to healthcare use.

**Methods:**

Self-reported demographic and health information from participants in the *45 and Up Study* cohort were linked to pharmaceutical claims from 2006–2009. Participants comprised 19,816 people with ≥1 opioid analgesic dispensing in the 12-months after recruitment to the cohort and 79,882 people not dispensed opioid analgesics. All participants were aged ≥45 years, were social security pharmaceutical beneficiaries, with no history of cancer. People dispensed opioid analgesics were classified as having acute (dispensing period <90 days), episodic (≥90 days and <3 ‘authority’ prescriptions for increased quantity supply) or long-term treatment (≥90 days and ≥3 authority prescriptions).

**Results:**

Of participants dispensed opioid analgesic 52% received acute treatment, 25% episodic treatment and 23% long-term treatment. People dispensed opioid analgesics long-term had an average of 14.9 opioid analgesic prescriptions/year from 2.0 doctors compared with 1.5 prescriptions from 1.1 doctors for people receiving acute treatment. People dispensed opioid analgesics reported more need-related factors such as poorer physical functioning and higher psychological distress. Long-term users were more likely to have access-related factors such as low-income and living outside major cities. After simultaneous adjustment, association with predisposing health factors and access diminished, but indicators of need such as osteoarthritis treatment, paracetamol use, and poor physical function were the strongest predictors for all opioid analgesic users.

**Conclusions:**

People dispensed opioid analgesics were in poorer health, reported higher levels of distress and poorer functioning than people not receiving opioid analgesics. Varying dispensing profiles were evident among people dispensed opioid analgesics for persistent pain, with those receiving episodic and long-term treatment dispensed the strongest opioid analgesics. The findings highlight the broad range of factors associated with longer term opioid analgesics use.

## Introduction

There is growing use of opioid analgesics for non-cancer pain, and corresponding apprehension about the safety and effectiveness of opioids [1]–[4]. In Australia there has been a marked increase in prescribing of oxycodone; prescribing rates rose by 152% between 2002–03 (35.3 per 1000 population) and 2007–08 (89.2 per 1000 population) [5], [6]. A recent study found that 43.9% of opioid analgesic prescribing were in people with persistent non-cancer pain (CNCP) and musculoskeletal conditions [7]. This prescribing is occurring in the context of significant gaps in the evidence base for long-term use of opioid analgesics in this setting of care.

Cohort studies have highlighted the realities of long-term opioid prescribing within population subgroups characterized by complex physical and psychological comorbidity, a phenomenon Sullivan [8] calls ‘adverse selection’. However, few studies have systematically studied other individual-level non-health factors in conjunction with these health factors, even though there is evidence that non-health factors are important drivers of healthcare use [9].

This study explores these aspects of treatment with opioid analgesics within a large population-based cohort, with the following specific aims:

1. To investigate patterns of publically-subsidised prescription opioid analgesic use for people with non-cancer pain in the community;
2. To explore the relationship between both health and non-health factors and patterns of prescription opioid analgesic use within the community.

## Materials and Methods

### Participants

Participants are from the *45 and Up Study*; a population-based cohort study in the Australian state of New South Wales (NSW). The *45 and Up Study* is the largest longitudinal cohort study in the Southern Hemisphere, and is designed to explore the determinants of the healthy ageing in the community aged ≥45 years [10]. The study has 267,151 participants; equivalent to 10% of the NSW population of this age. Participants were randomly sampled from the national health care database (Medicare Australia) and then mailed a baseline questionnaire between February 2006 and November 2008 (response rate 19%). This included consent to link questionnaire data with routinely collected health records held by the State and Commonwealth.

### Data sources and description

This study examined information reported by participants in the *45 and Up Study* baseline questionnaire linked with prescription claims after recruitment (i.e. prospective) and death records for these individuals. The *45 and Up Study* baseline questionnaire included a range of socio-demographic, behavioural, quality of life and clinical information.

The Australian Pharmaceutical Benefits Scheme (PBS) is Australia's universal pharmaceutical insurance scheme which entitles all residents to subsidized prescription medicines. Individuals make a co-payment toward the cost of each item dispensed and the remaining cost of is covered by the PBS [11]. Social security beneficiaries (e.g. aged pensioners, individuals with disabilities, the unemployed and other low income earners) pay a lower co-payment than the remainder of the community (‘general’ beneficiaries). In 2008 co-payments were AUD$5.00 for social security beneficiaries and $31.30 for general beneficiaries. Where the cost of the prescription medicines is lower than the co-payment, beneficiaries pay the lower amount. In Australia PBS subsidized medicines are commonly dispensed with one month's supply however the same co-payment is made irrespective of the dose or quantity dispensed.

Dispensings record included date of dispensing, beneficiary status, PBS item code [12], Anatomical Therapeutic Chemical (ATC) code, quantity supplied, and scrambled prescriber identifier. This study included all opioid analgesics (ATC N02A) listed on the PBS except those restricted to palliative care, dental procedures, and opioid maintenance therapy. For each item a morphine equivalent dose (MED) was derived based on type of opioid analgesic, strength, form, and quantity dispensed [13]. Dispensing records for each individual were used to derive the period of treatment (from first to last dispensing date +30 days), number of opioid analgesic prescriptions dispensed, number of increased supply dispensings, and total morphine equivalent dose. Date and fact of death for *45 and Up Study* participants were sourced from the state-based civil records (NSW Registry of Births, Deaths and Marriages).

### Inclusion and exclusion criteria

Prior to 1 April 2012 prescriptions falling under the general beneficiary co-payment (i.e. less than $31.30 in 2008) were not captured by the PBS. Many opioid analgesics in Australia are priced between $20 and $25 and were not recorded when dispensed to general beneficiaries. Approximately 86% of subsidised prescriptions in Australia are dispensed to social security beneficiaries [14]. Consequently, we restricted the study to participants with social security beneficiary status throughout the study period. To ensure opioid analgesics in this study were dispensed for non-cancer pain, we excluded participants who self-reported (45 and Up study) as ever being diagnosed with cancer (other than non-melanoma skin cancers). In this analysis we excluded persons who had been dispensed opioid analgesics restricted to subsidy for palliative care and any persons who had been dispensed opioid analgesics restricted to subsidy for opiate Veterans in Australia have access to pharmaceuticals under a separate funding scheme that provides higher benefits. These dispensings do not appear in our PBS data so we have excluded persons who self-report holding a Department of Veterans' Affairs (DVA) card as these persons have dispensings that are not recorded in the PBS data. Persons with missing essential details (overall n = 27; geographic location information (used to allocate remoteness status, n = 19) or self-report of language other than English (LOTE, n = 2), recent paracetamol use (n = 4), or help required for disability (n = 2)) were excluded because they created problems in model convergence with our strategy for missing covariates (missing as a category). We found estimating algorithm in PROC GENMOD (ridge stabilised Newton–Raphson) was unable to converge with very small cell sizes that resulted from treating missing as a category for these variables.

### Categorising opioid analgesic use

After restrictions, there were 99,698 participants included in the study. A follow-up period of 1 year of PBS data from date of enrolment in the *45 and Up Study* was used. Of these, 79,882 had no opioid analgesics dispensed during the study period, and 19,816 had ≥1 opioid analgesic dispensed. The first opioid dispensing in the 1 year of follow-up was considered the index dispensing. It is difficult to determine a standard supply period for opioid analgesics due to variation in pack size/volume, morphine equivalence, drug form, and daily dose. Patients also vary in their opioid analgesic tolerance, and the constancy and degree of their pain. Therefore cannot be assumed that a pack of 20 opioids analgesics represents a 20-day supply of analgesic therapy in the way than a pack of 20 tablets of an antihypertensive medication might. Most opioid analgesics are supplied in packs containing between 5–20, however PBS rules allow the dispensing of larger quantities per prescription to cover a one-month period where clinically needed [15]. Under these circumstances beneficiaries are charged only one co-payment, saving the individual several trips to the pharmacy each month for smaller quantities and the cost of multiple co-payments. Increased supply prescriptions are therefore an indicator of daily use over a one month period.

People dispensed opioid analgesics were categorised as receiving ‘acute’, ‘episodic’ or ‘long-term’ treatment based on their period of treatment and number of dispensings with increased supply. Treatment was classified as acute when opioid analgesics were dispensed over a period <90 days. Episodic treatment was classified as occurring over a period ≥90 days and with <3 increased quantity dispensings. Long-term treatment was classified as occurring ≥90 days and with ≥3 increased quantity dispensings. These categories are similar to those used in previous international studies [16], but have been adapted to the Australian PBS context. The utility of this classification system was confirmed with a group of pain management clinicians and pharmacists, and against the dataset [Table S1]. If there were several periods of opioid analgesic medicines use in the 1 year of follow-up that would lead to different classifications for the same individual, then the classification with the greatest period of treatment was used.

### Predictors of opioid analgesic dispensing

The Andersen-Newman model relates the individual characteristics of people to their healthcare utilization patterns, using a framework that relates use to three broad categories: the predisposition of an individual to use service; an individual's ability to access healthcare; and an individual's level of health needs [9].

In this study we examined the role of ‘predisposing-’, ‘access-’ and ‘need-’ related covariates which may predict dispensing of opioid analgesics. Predisposing factors included age, sex, relationship status (in relationship/previously in a relationship/never in a relationship), body mass index (healthy weight 18.5–<25 kg/m^2^/underweight <18.5 kg/m^2^/overweight 25–<30 kg/m^2^/obese 30–<35 kg/m^2^/morbidly obese 35+ kg/m^2^) education (10 years or less/12 years/post-secondary school/university degree), language other than English (LOTE) spoken at home (yes/no), number of physical activity sessions conducted per week (0–3/4–9/10–17/18+), smoking status (never smoker/current smoker/ex-smoker), alcohol consumption per week (0 drinks/1–4 drinks/5–9 drinks/10+ drinks), and sleep duration per day (0–6/7–8/9+ hours). Access-related factors examined were geographic remoteness using the ARIA+ score [17] (major city/inner regional/outer regional/remote), annual household income (<AUD$20K, $20–50k/$50–70K, $70K+/rather not say), private health insurance (public only/private), and work status (not working/part time/full time/retired for ill health). Need-related variables included self-rated health (excellent or good/poor or very poor), self-rated quality of life (excellent or good/poor or very poor), self-rated memory (excellent or good/poor or very poor), number of chronic conditions (0/1/2/3/4+), physical function score (Medical Outcomes Study Physical Function score [18] (0–19/20–39/40–59/60–89/90–100), help required with disability (yes/no), osteoarthritis treatments in the last month (yes/no), hip replacement in last 2 years (yes/no), knee replacement in last 2 years (yes/no), paracetamol use in past month (yes/no), treatment for anxiety or depression in last month (yes/no), and K10 Psychological Distress score [19] (low/moderate/high/very high).

Concomitant medicines use was defined as a dispensing of a selected medicine at any time during the individual's opioid analgesic supply period (any time within 30 days after a dispensing of an opioid analgesic medicine). We identified dispensings of PBS items falling under the following ATC codes: anticonvulsants (N03), antipsychotics (N05A), anxiolytics (N05B), laxatives (A06), analgesics (N02B), and antidepressants (N02C). We summarised the prescribing record for each participant with the number of opioid analgesic dispensings, the number of all dispensings, time from first to last supply in the defined temporal window, and the total number of opioid prescribers. An index of diversity [20] of the number and type of prescribers for each participant for opioid dispensings was calculated as:
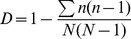
(1)where n = the total number of dispensings from an individual prescriber for a participant, and N = total number of dispensings of all prescribers for a participant. A dispensing pattern dominated by 1 or 2 prescribers has a lower diversity index than a pattern with many prescribers with relatively equal numbers of dispensings.

### Statistical Methods

All analysis was completed in SAS 9.2 (Cary, NC, USA). We found from an initial power analysis based on an early estimate of the numbers of participants in each treatment category that we had over 0.90 power (based on the two smallest categories n_1_ = 5000, n_2_ = 3200) to detect a 5% percentage point difference in rate (at 50% baseline rate, the most conservative), and also to be able to detect a difference of 1.5 in a continuous variable with a standard deviation of 20. The measures were summarized as means for continuous variables (e.g. total number of prescriptions); and rates for of co-prescribing, and dispensing of particular opioid types (many participants were dispensed several types of opioid analgesic so totals will sum to over 100%) according to type of opioid analgesic treatment. Comparison of these variables between groups was achieved by calculating 95% confidence intervals (normal distribution for continuous variables; binomial CIs for rates of co-prescribing and % dispensed each opioid type) for each summary measure.

Screening of measures associated with category of opioid user was undertaken by examining summary information (Table 1) and χ2 tests. All variables showed some association with category of opioid use, so each variable was modeled separately for each type of opioid user (compared to non-opioid users) with adjustments for age and sex. All variables showed strong associations with category of opioid use even with adjustment for age and sex so all of these variables were included in one model that simultaneously adjusted for all 27 predictor variables. All of these variables showed association with at least one category of opioid analgesic use so all of these were retained in the final fully adjusted model. The rate ratio and 95% CI for each association was estimated with a log-Poisson model with robust error variance [21].

**Table 1:** Personal characteristics of individuals (n, (% of treatment group)) dispensed opioid analgesics, by treatment group (Acute – supply <90 days; Episodic – Supply ≥90 days, <3 increased quantity dispensings; Long-term – Supply ≥90 days, ≥3 increased quantitative dispensings).

Category	Variable	Level	No Opioid user	Acute	Episodic	Long-term
**Predisposing**	Age	45–49 yrs	2,542 (3.2%)	446 (4.0%)	226 (4.5%)	192 (5.3%)
		50–54 yrs	3,610 (4.5%)	606 (5.4%)	338 (6.7%)	325 (9.0%)
		55–59 yrs	5,239 (6.6%)	831 (7.5%)	446 (8.8%)	391 (10.8%)
		60–64 yrs	10,210 (12.8%)	1,425 (12.8%)	652 (12.9%)	543 (15.0%)
		65–69 yrs	18,053 (22.6%)	2,349 (21.1%)	879 (17.4%)	596 (16.4%)
		70–74 yrs	15,330 (19.2%)	2,087 (18.7%)	875 (17.3%)	555 (15.3%)
		75–79 yrs	10,516 (13.2%)	1,469 (13.2%)	653 (12.9%)	402 (11.1%)
		80–84 yrs	10,321 (12.9%)	1,359 (12.2%)	663 (13.1%)	397 (11.0%)
		85+ yrs	4,061 (5.1%)	575 (5.2%)	313 (6.2%)	223 (6.2%)
	Sex	Male	35,965 (45.0%)	5,090 (45.7%)	2,139 (42.4%)	1,490 (41.1%)
		Female	43,917 (55.0%)	6,057 (54.3%)	2,906 (57.6%)	2,134 (58.9%)
	Marital Status	In relationship	52,393 (66.0%)	7,120 (64.3%)	2,856 (57.1%)	2,011 (56.0%)
		Previous relationship	20,203 (25.4%)	2,985 (27.0%)	1,610 (32.2%)	1,175 (32.7%)
		Never in relationship	6,791 (8.6%)	963 (8.7%)	535 (10.7%)	407 (11.3%)
	Education Level	10 years or less	36,675 (47.0%)	5,511 (50.7%)	2,686 (55.0%)	1,953 (55.7%)
		12 years	7,488 (9.6%)	973 (9.0%)	431 (8.8%)	319 (9.1%)
		Post secondary school	24,250 (31.1%)	3,316 (30.5%)	1,386 (28.4%)	982 (28.0%)
		University degree	9,572 (12.3%)	1,068 (9.8%)	383 (7.8%)	254 (7.2%)
	LOTE^1^ at home	No	71,538 (89.6%)	9,889 (88.7%)	4,480 (88.8%)	3,379 (93.2%)
		Yes	8,344 (10.4%)	1,258 (11.3%)	565 (11.2%)	245 (6.8%)
	PA sessions/week	0–3 sessions/week	14,121 (17.7%)	2,514 (22.6%)	1,412 (28.0%)	1,265 (34.9%)
		4–9 sessions/week	28,392 (35.5%)	3,771 (33.8%)	1,738 (34.4%)	1,140 (31.5%)
		10–17 sessions/week	24,147 (30.2%)	3,125 (28.0%)	1,209 (24.0%)	802 (22.1%)
		18+ sessions/week	13,222 (16.6%)	1,737 (15.6%)	686 (13.6%)	417 (11.5%)
	Body Mass Index	Underweight <18.5	1,216 (1.7%)	146 (1.4%)	96 (2.1%)	79 (2.4%)
		Healthy 18.5–<25	26,580 (36.4%)	2,933 (29.0%)	1,211 (26.7%)	840 (25.9%)
		Overweight 25–<30	29,086 (39.8%)	4,068 (40.3%)	1,609 (35.5%)	1,101 (33.9%)
		Obese 30–<35	11,588 (15.9%)	2,023 (20.0%)	1,046 (23.1%)	722 (22.2%)
		Morbidly Obese 35–<40	4,521 (6.2%)	930 (9.2%)	570 (12.6%)	506 (15.6%)
	Smoking Status	Never smoker	44,517 (55.9%)	5,597 (50.4%)	2,305 (45.9%)	1,497 (41.5%)
		Current smoker	5,291 (6.6%)	913 (8.2%)	624 (12.4%)	544 (15.1%)
		Ex smoker	29,787 (37.4%)	4,591 (41.4%)	2,090 (41.6%)	1,566 (43.4%)
	Alcohol consumption	0 drinks	31,668 (40.9%)	4,575 (42.4%)	2,282 (47.1%)	1,945 (56.1%)
		1–4 drinks	14,035 (18.1%)	1,913 (17.7%)	818 (16.9%)	487 (14.1%)
		5–9 drinks	12,860 (16.6%)	1,599 (14.8%)	684 (14.1%)	369 (10.6%)
		10+ drinks	18,860 (24.4%)	2,697 (25.0%)	1,056 (21.8%)	665 (19.2%)
	Hours sleeping (per day)	0–6 hrs	12,161 (15.8%)	2,033 (19.0%)	1,136 (23.7%)	804 (23.5%)
		7–8 hrs	55,669 (72.5%)	7,282 (68.2%)	2,897 (60.5%)	1,815 (53.1%)
		9+ hrs	8,947 (11.7%)	1,367 (12.8%)	754 (15.8%)	796 (23.3%)
**Access**	Remoteness Index	Major city	33,214 (41.6%)	4,496 (40.3%)	2,097 (41.6%)	1,200 (33.1%)
		Inner regional	29,701 (37.2%)	4,242 (38.1%)	1,813 (35.9%)	1,557 (43.0%)
		Outer regional	15,392 (19.3%)	2,177 (19.5%)	991 (19.6%)	780 (21.5%)
		Remote	1,575 (2.0%)	232 (2.1%)	144 (2.9%)	87 (2.4%)
	Household Income^2^	<$20k/year	29,080 (39.3%)	4,453 (43.6%)	2,408 (53.2%)	1,825 (56.4%)
		$20–50k/year	24,933 (33.7%)	3,156 (30.9%)	1,118 (24.7%)	748 (23.1%)
		$50–70/year	3,580 (4.8%)	397 (3.9%)	107 (2.4%)	60 (1.9%)
		$70k+/year	1,720 (2.3%)	194 (1.9%)	43 (1.0%)	26 (0.8%)
		Rather not say	14,591 (19.7%)	2,002 (19.6%)	849 (18.8%)	577 (17.8%)
	Work status	Not working	55,371 (70.8%)	7,254 (66.4%)	3,024 (61.1%)	1,851 (52.1%)
		Part time	8,963 (11.5%)	1,131 (10.3%)	381 (7.7%)	224 (6.3%)
		Full time	3,853 (4.9%)	491 (4.5%)	135 (2.7%)	63 (1.8%)
		Retired for ill health	10,071 (12.9%)	2,053 (18.8%)	1,412 (28.5%)	1,415 (39.8%)
	Private Health Insurance	Private	40,494 (50.7%)	5,197 (46.6%)	1,870 (37.1%)	1,225 (33.8%)
		None	39,388 (49.3%)	5,950 (53.4%)	3,175 (62.9%)	2,399 (66.2%)
**Need**	Self-rated Health	Excellent or Good	63,250 (83.0%)	7,800 (73.8%)	2,686 (56.7%)	1,536 (45.1%)
		Poor or very poor	12,980 (17.0%)	2,765 (26.2%)	2,049 (43.3%)	1,866 (54.9%)
	Self-rated QOL^3^	Excellent or Good	64,363 (86.7%)	8,225 (80.1%)	3,037 (66.4%)	1,816 (54.9%)
		Poor or very poor	9,862 (13.3%)	2,038 (19.9%)	1,537 (33.6%)	1,493 (45.1%)
	Self-rated Memory	Excellent or Good	59,562 (77.8%)	7,954 (74.8%)	3,359 (70.1%)	2,270 (66.0%)
		Poor or very poor	16,970 (22.2%)	2,676 (25.2%)	1,430 (29.9%)	1,168 (34.0%)
	# of chronic conditions	0 conditions	14,394 (18.0%)	1,790 (16.1%)	701 (13.9%)	427 (11.8%)
		1 condition	22,912 (28.7%)	2,883 (25.9%)	1,108 (22.0%)	695 (19.2%)
		2 conditions	21,007 (26.3%)	2,945 (26.4%)	1,223 (24.2%)	900 (24.8%)
		3 conditions	12,760 (16.0%)	1,941 (17.4%)	1,017 (20.2%)	734 (20.3%)
		4+ conditions	8,809 (11.0%)	1,588 (14.2%)	996 (19.7%)	868 (24.0%)
	Physical Function Score^4^	MOSPF 0–19	3,615 (5.2%)	929 (9.6%)	795 (18.1%)	928 (28.8%)
		MOSPF 20–39	4,345 (6.2%)	1,087 (11.2%)	816 (18.6%)	792 (24.6%)
		MOSPF 40–59	6,772 (9.7%)	1,344 (13.9%)	829 (18.9%)	586 (18.2%)
		MOSPF 60–89	22,480 (32.3%)	3,133 (32.3%)	1,210 (27.6%)	681 (21.1%)
		MOSPF 90–100	32,430 (46.6%)	3,207 (33.1%)	733 (16.7%)	235 (7.3%)
	Help with disability	No	74,525 (93.3%)	9,917 (89.0%)	3,998 (79.2%)	2,366 (65.3%)
		Yes	5,357 (6.7%)	1,230 (11.0%)	1,047 (20.8%)	1,258 (34.7%)
	Osteoarthritis treatment In last month	No	71,608 (89.6%)	9,351 (83.9%)	3,727 (73.9%)	2,353 (64.9%)
		Yes	8,274 (10.4%)	1,796 (16.1%)	1,318 (26.1%)	1,271 (35.1%)
	Recent (<2 yrs) hip replacement surgery	No	79,506 (99.5%)	11,065 (99.3%)	4,998 (99.1%)	3,581 (98.8%)
		Yes	376 (0.5%)	82 (0.7%)	47 (0.9%)	43 (1.2%)
	Recent (<2 yrs) knee replacement surgery	No	79,210 (99.2%)	10,970 (98.4%)	4,943 (98.0%)	3,557 (98.2%)
		Yes	672 (0.8%)	177 (1.6%)	102 (2.0%)	67 (1.8%)
	Used paracetamol in last month	No	60,254 (75.4%)	7,362 (66.0%)	2,649 (52.5%)	1,770 (48.8%)
		Yes	19,628 (24.6%)	3,785 (34.0%)	2,396 (47.5%)	1,854 (51.2%)
	Anxiety treatment in last month	No	65,745 (95.4%)	9,093 (93.9%)	3,930 (89.6%)	2,832 (87.0%)
		Yes	3,202 (4.6%)	594 (6.1%)	457 (10.4%)	424 (13.0%)
	Depression treatment in last month	No	64,046 (92.9%)	8,734 (90.2%)	3,679 (83.9%)	2,549 (78.3%)
		Yes	4,901 (7.1%)	953 (9.8%)	708 (16.1%)	707 (21.7%)
	K10 Psychological	Low	61,370 (80.0%)	7,910 (74.0%)	3,056 (63.2%)	1,917 (55.5%)
	Distress	Moderate	9,899 (12.9%)	1,659 (15.5%)	940 (19.4%)	758 (21.9%)
		High	3,715 (4.8%)	749 (7.0%)	527 (10.9%)	477 (13.8%)
		Very High	1,687 (2.2%)	372 (3.5%)	313 (6.5%)	305 (8.8%)
	**Total**		79,882	11,147	5,045	3,264

1 Language other than English.

2 Household Income in Australian Dollars.

3 Quality of Life.

4 Medical-outcomes study physical function scale, values range from 0 (lowest physical function level) to 100 (highest physical function level.

### Ethics Information

The 45 and Up Study has approval from the University of NSW Ethics Committee, and this study was approved by The NSW Population and Health Services Research Ethics Committee and the Department of Health and Ageing Departmental Ethics Committee. Participants in The 45 and Up study gave written consent to use of their questionnaire information to be used for research, and to link their information to their PBS data.

## Results

**Figure 1:**
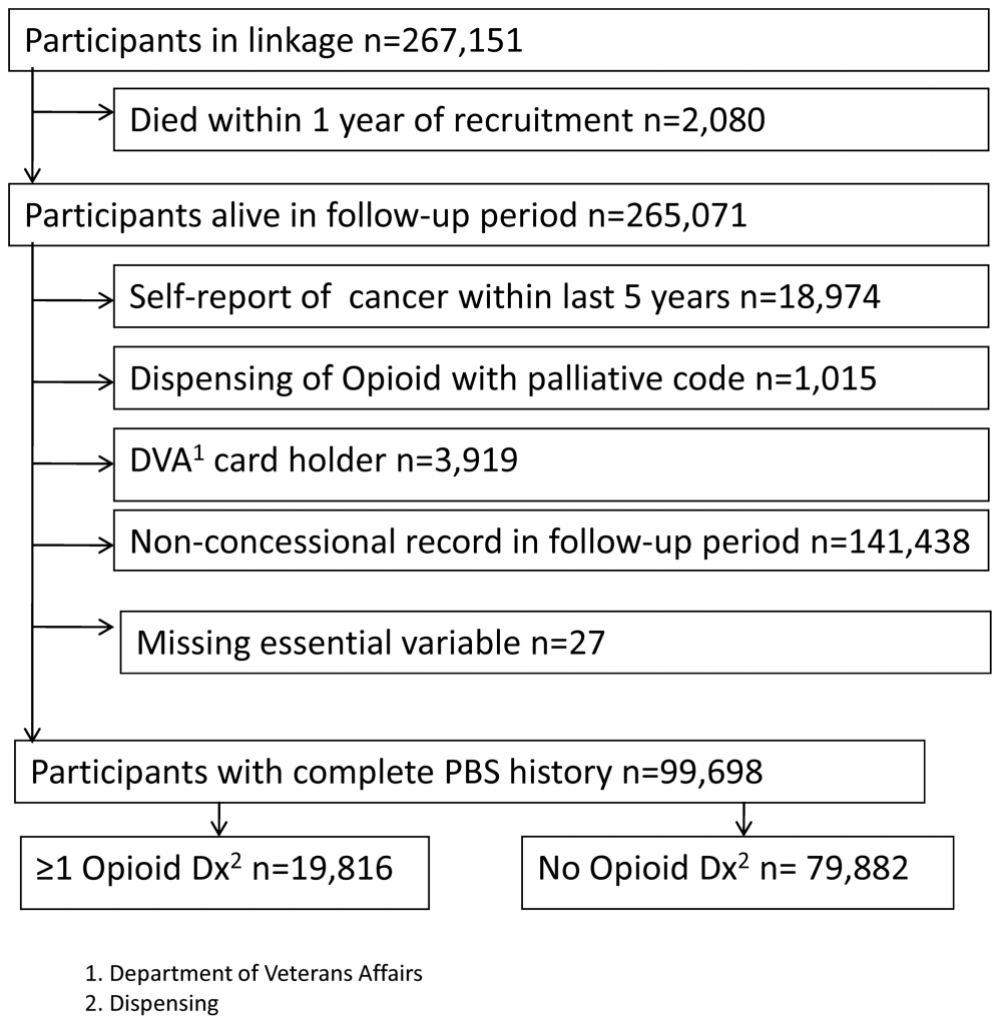
Participant flow according to inclusion and exclusions rules in this study.

A total of 99,698 participants met the inclusion criteria for the study and had complete PBS medicines dispensing records for the index period (Figure 1). Of these, 19,816 (19.8%) had one or more opioid analgesic dispensed during the study period. Over half of this group (51.6%, n = 11,147) received acute treatment, 26.3% (n = 5045) received episodic treatment and 21.8% (n = 3264) long-term treatment.

The characteristics of participants who were dispensed opioid analgesics are shown in Table 1. Individuals receiving acute treatment had a small number of opioid analgesics dispensings (mean 1.45) over a short period (44.5 days). Individuals receiving episodic treatment had a higher number of opioid analgesic dispensings (mean 7.4) over a longer time (240 days) (Table 2), while long-term opioid analgesic users had the highest number of opioid analgesic dispensings (mean 14.9) with the average supply period 328.9 days; close to the entire available follow-up period of 1 year. The acute group typically had only 1 opioid analgesic prescriber (average 1.2) compared to higher total number of prescribers in the episodic (2.2) and long-term (2.3) groups. This was reflected in the low index of diversity of prescribers for the participants classified as acute users of opioid analgesics (Table 2). The episodic and long-term groups had similar number of prescribers and diversity index scores. Overall the extent of co-prescribing of contraindicated drug classes with opioid analgesics (antipsychotics, anxiolytics, anticonvulsants) was low (<1%) and similar across treatment groups. Few individuals had co-prescribing of laxatives with opioid analgesics (<1% across all groups) or PBS analgesics was (<4% across all groups). The long-term opioid analgesic treatment groups were more likely (6.3%) to have been dispensed an antidepressant medication during the supply period for an opioid analgesic than the other groups (4.1% acute and 4.9% episodic).

**Table 2:** Characteristics of opioid analgesic dispensings, including mean number, mean time of opioid supply, mean number of prescribers, mean index of diversity of opioid prescribers (higher index = more prescribers and more variance in number of dispensings per prescriber), mean oral morphine equivalents per day during period of opioid dispensings, and co-dispensing of other medicines during period of opioid supply by treatment group (see methods for details).

	Opioid analgesic treatment		
	Acute	Episodic	Long-term
**Opioid analgesic dispensings**	1.45 (1.41–1.49)	7.4 (7.2–7.7)	14.9 (14.5–15.3)
**All dispensings**	44.5 (44.0–45.1)	65.3 (64.2–66.4)	81.6 (80.2–83.1)
**Days from first dispensing to end of supply**	52.7 days (52.1–53.4)	240.1 (237.6–242.7)	328.9 (326.8–331.1)
**Mean number of opioid prescribers**	1.13 (1.12–1.14)	1.87 (1.84–1.9)	2.03 (1.98–2.08)
**Index of diversity of opioid prescribers**	0.053 (0.05–0.056)	0.249 (0.242–0.256)	0.200 (0.193–0.207)
**Mean MED:**	6.0 (5.8–6.3)	11.3 (10.4–12.1)	59.4 (55.9–62.8)
**Co-prescribing between OA dispensings**			
Anticonvulsants (ATC: N03)	0.47% (0.1%–0.9%)	0.46% (0.3%–0.6%)	0.73% (0.4%–1%)
Antipsychotics (ATC: N05A)	0.3% (0%–0.6%)	0.43% (0.2%–0.6%)	0.34% (0.1%–0.5%)
Anxiolytics (ATC: N05B)	0.5% (0.1%–0.9%)	0.4% (0.2%–0.6%)	1.7% (1.3%–2.1%)
Laxatives (ATC: A06)	0.79% (0.3%–1.3%)	0.64% (0.4%–0.9%)	0.86% (0.5%–1.2%)
Analgesics (ATC: N02B)	3.2% (2.2%–4.2%)	3.8% (3.3%–4.3%)	3.2% (2.6%–3.8%)
Antidepressants (ATC: N02C)	4.1% (3%–5.2%)	4.9% (4.3%–5.5%)	6.3% (5.5%–7.1%)

Codeine was the most commonly dispensed opioid analgesic (Table 3) across all treatment groups and was dispensed most often for the acute and episodic groups (68.5% and 67.7%, respectively). The amount of codeine dispensed was greatest in users in the acute treatment group (5.1 mg/day MED) compared with the episodic group (1.8 mg/day MED). Codeine was dispensed less commonly to participants in the long-term opioid analgesic treatment group (56.6%).

**Table 3:** Use of opioid analgesics by drug class and treatment group, with total morphine equivalent dose (/day across entire opioid supply period) for those dispensed type at least once.

Opioid Class	% of treatment group dispensed medicines in drug class			Morphine Equivalent Dose (mg/day) for treated participants		
	Acute	Episodic	Long-term	Acute	Episodic	Long-term
Morphine	1.4% (0.7%–2.1%)	3.9% (3.4%–4.4%)	10.4% (9.4%–11.4%)	17.3 (12.3–22.3)	18.9 (13.7–24.1)	73.7 (61.1–86.2)
Methadone	0%	0.2% (0.1%–0.3%)	1.7% (1.3%–2.1%)	0	36.5 (6.3–66.6)	340.8 (254.4–427.2)
Tramadol	15.6% (13.5%–17.7%)	32% (30.7%–33.3%)	33.4% (31.8%–35%)	14.6 (13.6–15.7)	8.2 (7.8–8.7)	37.3 (35.8–38.8)
Oxycodone	18.2% (16%–20.4%)	28.3% (27.1%–29.5%)	32.1% (30.5%–33.7%)	11.6 (10.6–12.6)	6.9 (5.8–7.9)	37.9 (33.3–42.5)
Fentanyl	1.1% (0.5%–1.7%)	4.2% (3.6%–4.8%)	7.8% (6.9%–8.7%)	17.6 (13.6–21.7)	13.5 (11–16.1)	23.9 (20.6–27.2)
Hydromorphone	0%	0.1% (0.01%–0.2%)	0.1% (0%–0.2%)	0	26.2 (−19–71.4)	53.9 (−46.3–154.1)
Codeine	68.5% (65.8%–71.2%)	67.7% (66.4%–69%)	56.6% (54.9%–58.3%)	5.7 (5.4–5.9)	2.0 (1.9–2.1)	13.2 (12.8–13.7)
Buprenorphine	3.2% (2.2%–4.2%)	9.7% (8.9%–10.5%)	15.9% (14.6%–17.2%)	33.2 (29.3–37.2)	29.1 (25.3–32.8)	54.6 (50.5–58.8)

Dispensing of oxycodone, tramadol, and fentanyl was more common among the episodic and long-term opioid analgesic treatment groups than the acute group, although the acute group were dispensed tramadol and oxycodone in larger amounts (MED/day) than the episodic group (Table 3). Morphine was typically only dispensed to the long-term treatment group (10.4% vs. 1.4/3.9% in acute/episodic groups) and in greater quantities. Buprenorphine was also not commonly dispensed to acute users (3.2%) and more commonly dispensed to episodic (9.7%) and long-term users (15.9%). Dispensing of hydromorphone and methadone was rare (<2% across all treatment groups), with those dispensed methadone in the long-term group notable for the high dose they were dispensed (341 mg/day MED).

Table 4 shows the socio-demographic and health characteristics of the three treatment groups, adjusted for age and sex. All opioid analgesic users were likely to be younger than the group not dispensed opioid analgesics, with the episodic and long-term groups much less likely to be aged 70 to 84 years than participants without dispensings of opioid analgesics. All treatment groups dispensed opioid analgesics were more likely to report less physical activity, higher body mass, and being current or ex-smokers than those not dispensed opioid analgesics. The strength of association between these predictors and opioid analgesic dispensing was correlated with the most intense pattern of dispensing, e.g. the acute group were 1.26 (Rate Ratio, 95% CI 1.18–1.35) times more likely to be current smokers than those not dispensed opioid analgesics, and the episodic (Rate Ratio 2.08; 1.90–2.27) and long-term users (Rate Ratio 2.51; 2.27–2.67). Rates of high levels of alcohol use (10+ drinks per week) showed a different pattern, they were similar between those not dispensed opioid analgesics and the acute treatment group (Rate Ratio 0.99; 0.94–1.03); but episodic (Rate Ratio 0.83; 0.77–0.89) and long-term users (Rate Ratio, 0.61; 0.56–.067) were much less likely to report higher level of alcohol use.

**Table 4:** Rate-ratio (RR) of the association of variables (by Andersen-Newman model categories of Predisposing, Access, and Need) belonging to an opioid analgesic treatment group (Acute, Episodic, Long-term – see Methods and Materials for details) with those not dispensed an opioid analgesic as the reference group, each variable adjusted for age and sex.

Category	Variable	Level	Acute	Episodic	Long-term
**Predisposing**	Age	45–49 yrs	Reference	Reference	Reference
		50–54 yrs	0.96 (0.86–1.08)	1.05 (0.89–1.23)	1.18 (0.99–1.4)
		55–59 yrs	0.92 (0.82–1.02)	0.96 (0.83–1.12)	0.99 (0.84–1.17)
		60–64 yrs	0.82 (0.74–0.9)	0.74 (0.64–0.85)	0.72 (0.61–0.84)
		65–69 yrs	0.77 (0.7–0.84)	0.57 (0.5–0.66)	0.46 (0.39–0.54)
		70–74 yrs	0.8 (0.72–0.88)	0.67 (0.58–0.77)	0.5 (0.43–0.59)
		75–79 yrs	0.81 (0.74–0.9)	0.73 (0.63–0.84)	0.53 (0.45–0.63)
		80–84 yrs	0.77 (0.7–0.85)	0.75 (0.65–0.87)	0.54 (0.45–0.63)
		85+ yrs	0.83 (0.74–0.93)	0.88 (0.75–1.04)	0.74 (0.62–0.9)
	Sex	Male	Reference	Reference	Reference
		Female	0.96 (0.93–1)	1.07 (1.01–1.13)	1.08 (1.01–1.15)
	Relationship status	In relationship	Reference	Reference	Reference
		Previously in relationship	1.08 (1.04–1.12)	1.37 (1.29–1.46)	1.42 (1.32–1.53)
		Never in relationship	0.99 (0.93–1.05)	1.28 (1.17–1.41)	1.27 (1.15–1.41)
	Education	10 years or less	Reference	Reference	Reference
		12 years	0.87 (0.81–0.93)	0.78 (0.71–0.86)	0.78 (0.7–0.88)
		Post secondary school	0.91 (0.87–0.94)	0.8 (0.75–0.85)	0.78 (0.72–0.84)
		University degree	0.76 (0.71–0.81)	0.57 (0.51–0.63)	0.52 (0.46–0.59)
	LOTE at home^1^	No	Reference	Reference	Reference
		Yes	1.06 (1–1.12)	1.03 (0.95–1.12)	0.59 (0.52–0.67)
	PA sessions per week	18+ sessions/week	Reference	Reference	Reference
		10–17 sessions/week	1.32 (1.25–1.4)	1.82 (1.66–1.99)	2.73 (2.45–3.04)
		4–9 sessions/week	1.03 (0.97–1.08)	1.18 (1.08–1.28)	1.31 (1.17–1.46)
		0–3 sessions/week	1 (0.95–1.06)	0.98 (0.89–1.07)	1.09 (0.97–1.23)
	Body Mass	Healthy 18.5–<25	Reference	Reference	Reference
	Index	Underweight <18.5	1.06 (0.91–1.24)	1.57 (1.29–1.92)	1.84 (1.47–2.31)
		Overweight25–<30	1.24 (1.19–1.3)	1.25 (1.16–1.35)	1.24 (1.13–1.35)
		Obese 30–<35	1.5 (1.43–1.59)	1.97 (1.81–2.13)	1.92 (1.74–2.11)
		Morbidly Obese 35+	1.71 (1.6–1.83)	2.57 (2.34–2.84)	3.04 (2.72–3.38)
	Smoking Status	Never smoker	Reference	Reference	Reference
		Current smoker	1.26 (1.18–1.35)	2.08 (1.9–2.27)	2.51 (2.27–2.76)
		Ex smoker	1.2 (1.16–1.25)	1.41 (1.33–1.5)	1.65 (1.53–1.77)
	Alcohol consumption (drinks per week)	0 drinks	Reference	Reference	Reference
		1–4 drinks	0.95 (0.9–1)	0.84 (0.78–0.91)	0.59 (0.54–0.66)
		5–9 drinks	0.88 (0.84–0.93)	0.78 (0.72–0.85)	0.52 (0.46–0.58)
		10+ drinks	0.99 (0.94–1.03)	0.83 (0.77–0.89)	0.61 (0.56–0.67)
		7–8 hrs	0.95 (0.89–1.02)	0.71 (0.64–0.79)	0.52 (0.47–0.59)
	Hours sleeping (per day)	0–6 hrs	1.23 (1.18–1.29)	1.68 (1.57–1.79)	1.88 (1.74–2.04)
		7–8 hrs	Reference	Reference	Reference
		9+ hrs	1.14 (1.08–1.21)	1.55 (1.44–1.68)	2.6 (2.4–2.82)
**Access**	Remoteness	Major city	Reference	Reference	Reference
	Index	Inner regional	1.05 (1–1.09)	0.98 (0.92–1.04)	1.42 (1.32–1.53)
		Outer regional	1.03 (0.98–1.08)	1.02 (0.94–1.09)	1.34 (1.23–1.47)
		Remote	1.05 (0.93–1.19)	1.37 (1.17–1.62)	1.39 (1.13–1.72)
	Household	<$20k/year	Reference	Reference	Reference
	Income^2^	$20–50k/year	0.84 (0.81–0.88)	0.57 (0.53–0.61)	0.5 (0.46–0.55)
		$50–70/year	0.75 (0.68–0.83)	0.39 (0.32–0.48)	0.29 (0.22–0.37)
		$70k+/year	0.75 (0.66–0.86)	0.33 (0.24–0.44)	0.25 (0.17–0.37)
		Rather not say	0.92 (0.87–0.96)	0.73 (0.68–0.79)	0.66 (0.6–0.72)
	Private Health	None	Reference	Reference	Reference
	Insurance	Private	0.89 (0.85–0.92)	0.62 (0.59–0.66)	0.56 (0.52–0.6)
	Work Status	Not working	Reference	Reference	Reference
		Part time	0.91 (0.86–0.97)	0.71 (0.63–0.79)	0.59 (0.51–0.68)
		Full time	0.9 (0.82–0.98)	0.57 (0.47–0.68)	0.37 (0.29–0.48)
		Retired due to ill health	1.45 (1.38–1.52)	2.42 (2.27–2.57)	3.71 (3.46–3.98)
**Need**	Self-rated	Excellent or Good	Reference	Reference	Reference
	Health	Poor or very poor	1.58 (1.52–1.65)	3.26 (3.08–3.45)	5.06 (4.73–5.41)
	Self-rated	Excellent or Good	Reference	Reference	Reference
	QOL^2^	Poor or very poor	1.49 (1.42–1.56)	2.88 (2.71–3.06)	4.49 (4.19–4.81)
	Self-rated	Excellent or Good	Reference	Reference	Reference
	memory	Poor or very poor	1.15 (1.1–1.19)	1.42 (1.34–1.51)	1.71 (1.6–1.83)
	# of Chronic	0 conditions	Reference	Reference	Reference
	Conditions	1 condition	1.03 (0.97–1.09)	1.04 (0.95–1.14)	1.11 (0.99–1.25)
		2 conditions	1.14 (1.08–1.2)	1.26 (1.15–1.38)	1.6 (1.43–1.8)
		3 conditions	1.22 (1.15–1.3)	1.7 (1.54–1.87)	2.14 (1.9–2.41)
		4+ conditions	1.42 (1.33–1.51)	2.35 (2.13–2.58)	3.58 (3.19–4.01)
	Physical-function Score^3^	MOSPF 90–100	Reference	Reference	Reference
		MOSPF 60–89	1.4 (1.34–1.47)	2.41 (2.2–2.64)	4.42 (3.81–5.12)
		MOSPF 40–59	1.92 (1.81–2.03)	5.14 (4.66–5.66)	11.97 (10.3–13.9)
		MOSPF 20–39	2.34 (2.19–2.49)	7.49 (6.8–8.25)	23.04 (19.97–26.59)
		MOSPF 0–19	2.43 (2.27–2.6)	8.8 (7.98–9.71)	32.12 (27.9–36.98)
	Help with disability	No	Reference	Reference	Reference
		Yes	1.58 (1.5–1.67)	3.09 (2.89–3.3)	5.92 (5.54–6.33)
	Osteoarthritis Treatment^4^	No	Reference	Reference	Reference
		Yes	1.59 (1.52–1.67)	2.85 (2.68–3.02)	4.47 (4.18–4.77)
	Recent^5^ hip replacement surgery	No	Reference	Reference	Reference
		Yes	1.49 (1.23–1.82)	1.93 (1.47–2.52)	2.59 (1.95–3.45)
	Recent^5^ knee replacement surgery	No	Reference	Reference	Reference
		Yes	1.75 (1.53–1.99)	2.32 (1.93–2.79)	2.3 (1.83–2.9)
	Used paracetamol^4^	No	Reference	Reference	Reference
		Yes	1.5 (1.45–1.56)	2.58 (2.45–2.73)	2.98 (2.79–3.18)
	Anxiety treatment^4^	No	Reference	Reference	Reference
		Yes	1.24 (1.15–1.34)	2.05 (1.87–2.25)	2.4 (2.17–2.65)
	Depression Treatment^4^	No	Reference	Reference	Reference
		Yes	1.31 (1.23–1.4)	2.19 (2.02–2.37)	2.85 (2.62–3.1)
	K10 Psychological	Low	Reference	Reference	Reference
	Distress	Moderate	1.24 (1.18–1.31)	1.81 (1.68–1.94)	2.21 (2.03–2.4)
		High	1.43 (1.34–1.54)	2.55 (2.33–2.8)	3.37 (3.05–3.72)
		Very high	1.53 (1.39–1.68)	3.19 (2.85–3.57)	4.41 (3.92–4.96)

1 Language other than English.

2 Quality of Life.

3 Medical-outcomes study physical function scale, values range from 0 (lowest physical function level) to 100 (highest physical function level.

4 Self-reported, in last 28 days.

5 Self-reported surgery within 2 years of baseline recruitment.

For access-related factors, the episodic and long-term opioid analgesic treatment groups were more likely to reside in a remote area (Rate Ratios 1.37; 1.17–1.62 and 1.39; 1.13–172 respectively) than those not dispensed opioid analgesics. Individuals with higher household incomes were less likely to be dispensed opioid analgesics in any treatment group, with fewer individuals in the episodic and long-term treatment groups having higher household income. Those dispensed opioid analgesics were also less likely to report having any type of private health insurance across all treatment groups. Participants dispensed opioid analgesics were also less likely to be in either full or part-time work, with the rate decreasing with intensity of treatment. Individuals in the episodic and long-term groups were much more likely to report retiring due to ill health (Rate Ratios 2.42; 2.27–2.67 and 3.71; 3.46–3.98, respectively) than those not dispensed opioid analgesics.

For need factors, all treatment groups showed higher health-related needs than those not dispensed opioid analgesics, with higher need occurring with treatment intensity(from acute to long-term use). Individuals in the long-term treatment group were much more likely to report lower self-rated health (Rate Ratio 5.06; 4.73–5.41) and self-rated quality of life (Rate Ratios 4.49; 4.19–4.81) than those not dispensed opioid analgesics. Individuals dispensed opioid analgesics were more likely to report higher numbers of chronic conditions and lower physical function – the long-term treatment group were 32.1 (Rate Ratio 27.9–36.9) times more likely to report the lowest level of physical function than those not dispensed opioid analgesics, and also report needing help with a disability. Participants dispensed opioid analgesics were also more likely to report recent joint replacement surgery and paracetamol use than those not dispensed opioid analgesics. All treatment groups were more likely than those not dispensed opioid analgesics to report recent treatment for anxiety and depression, with the episodic and long-term groups the most likely to report treatment for these conditions. These two treatment groups were also much more likely to have very high levels of psychological distress (Rate Ratios 3.19; 2.85–3.57 and 4.41; 3.92–4.96 respectively) than those not dispensed opioid analgesics.

In models that adjusted for all predictor variables (Table 5), physical functioning maintained the strongest relationship with all three opioid analgesic treatment groups – the episodic and long-term groups were 3.1 (2.7–3.5) and 7.3 (6.2–8.8) times more likely to have the lowest level of physical function (MOS-PF 0–19) than those not dispensed opioid analgesics. Risk ratios for several other variables were lower in the full-adjusted model but remained significantly associated across all treatment groups; in particular, current or ex- smoker status, retirement due to ill health, poorer self-rated health, needing help with a disability, recent osteoarthritis treatment, and recent paracetamol use. Recent osteoarthritis treatment in the acute, episodic, and long-term groups (RR 1.23; 1.18–1.30, 1.56; 1.47–1.66, 1.95; 1.82–2.09, respectively) and paracetamol use in the last month (RR 1.29; 1.24–1.34, 1.8; 1.70–1.90, 1.7; 1.59–1.82) were the other two strongest predictors of opioid analgesic dispensing.

**Table 5:** Rate-ratio (RR) of the association of variables (by Andersen-Newman model categories of Predisposing, Access, and Need) belonging to a category of opioid use (Acute, Episodic, Long-term – see Methods and Materials for details) with non-users as the reference group, each variable fully adjusted by all variables in this table.

Category	Variable	Level	Acute	Episodic	Long-term
**Predisposing**	Age	45–49 yrs	Reference	Reference	Reference
		50–54 yrs	0.9 (0.8–1)	0.96 (0.82–1.12)	0.95 (0.81–1.11)
		55–59 yrs	0.83 (0.75–0.92)	0.82 (0.71–0.96)	0.75 (0.64–0.88)
		60–64 yrs	0.77 (0.69–0.85)	0.75 (0.65–0.87)	0.68 (0.58–0.79)
		65–69 yrs	0.78 (0.71–0.86)	0.76 (0.66–0.88)	0.62 (0.53–0.73)
		70–74 yrs	0.79 (0.72–0.88)	0.82 (0.71–0.95)	0.64 (0.54–0.75)
		75–79 yrs	0.78 (0.7–0.87)	0.8 (0.69–0.93)	0.56 (0.48–0.67)
		80–84 yrs	0.72 (0.64–0.8)	0.75 (0.64–0.87)	0.5 (0.42–0.6)
		85+ yrs	0.73 (0.64–0.82)	0.73 (0.61–0.88)	0.52 (0.42–0.63)
	Sex	Male	Reference	Reference	Reference
		Female	0.92 (0.88–0.96)	0.92 (0.86–0.98)	0.91 (0.85–0.98)
	Relationship status	In relationship	Reference	Reference	Reference
		Previously in relationship	0.99 (0.95–1.04)	1.04 (0.98–1.11)	1.02 (0.95–1.1)
		Never in relationship	0.94 (0.88–1)	1.03 (0.94–1.13)	0.96 (0.86–1.06)
	Education	10 years or less	Reference	Reference	Reference
		12 years	0.93 (0.87–0.99)	0.95 (0.86–1.05)	1.08 (0.97–1.21)
		Post secondary school	0.96 (0.92–1)	0.94 (0.88–1)	0.96 (0.89–1.03)
		University degree	0.87 (0.81–0.93)	0.83 (0.75–0.92)	0.89 (0.78–1)
	LOTE^1^ at home	No	Reference	Reference	Reference
		Yes	1.06 (1–1.12)	0.94 (0.86–1.03)	0.58 (0.51–0.66)
	PA sessions per week	18+ sessions/week	Reference	Reference	Reference
		10–17 sessions/week	1.06 (1–1.13)	1.06 (0.96–1.16)	1.12 (1.01–1.25)
		4–9 sessions/week	0.95 (0.9–1.01)	0.98 (0.9–1.07)	1.02 (0.91–1.13)
		0–3 sessions/week	0.99 (0.94–1.05)	0.96 (0.88–1.05)	1.07 (0.96–1.19)
	Body Mass	Healthy 18.5–<25	Reference	Reference	Reference
	Index	Underweight <18.5	0.99 (0.85–1.16)	1.24 (1.02–1.5)	1.27 (1.02–1.57)
		Overweight 25–<30	1.18 (1.13–1.23)	1.15 (1.07–1.23)	1.1 (1.01–1.19)
		Obese 30–<35	1.28 (1.21–1.35)	1.38 (1.27–1.5)	1.18 (1.08–1.3)
		Morbidly Obese >35	1.29 (1.2–1.38)	1.34 (1.22–1.48)	1.19 (1.07–1.32)
	Smoking Status	Never smoker	Reference	Reference	Reference
		Current smoker	1.16 (1.08–1.24)	1.5 (1.37–1.64)	1.62 (1.47–1.79)
		Ex smoker	1.13 (1.09–1.18)	1.2 (1.13–1.28)	1.32 (1.23–1.42)
	Alcohol consumption	0 drinks	Reference	Reference	Reference
		1–4 drinks	1.02 (0.97–1.07)	1.03 (0.95–1.11)	0.83 (0.76–0.92)
		5–9 drinks	0.99 (0.93–1.04)	1.06 (0.98–1.15)	0.8 (0.72–0.89)
		10+ drinks	1.06 (1.01–1.12)	1.04 (0.96–1.12)	0.84 (0.77–0.92)
	Hours sleeping (per day)	0–6 hrs	1.13 (1.08–1.18)	1.28 (1.2–1.37)	1.31 (1.21–1.41)
		7–8 hrs	Reference	Reference	Reference
		9+ hrs	0.96 (0.9–1.01)	0.99 (0.92–1.07)	1.29 (1.19–1.39)
**Access**	Remoteness	Major city	Reference	Reference	Reference
	Index	Inner regional	1.05 (1–1.09)	0.97 (0.91–1.03)	1.28 (1.19–1.37)
		Outer regional	1.03 (0.98–1.08)	0.99 (0.92–1.07)	1.21 (1.11–1.32)
		Remote	1.02 (0.9–1.15)	1.24 (1.05–1.45)	1.18 (0.96–1.45)
	Household income	<$20k/year	Reference	Reference	Reference
		$20–50k/year	0.96 (0.92–1.01)	0.87 (0.81–0.94)	0.89 (0.82–0.97)
		$50–70/year	0.93 (0.84–1.03)	0.79 (0.65–0.96)	0.79 (0.62–1.01)
		$70k+/year	0.96 (0.84–1.11)	0.72 (0.53–0.97)	0.86 (0.59–1.25)
		Rather not say	1 (0.95–1.05)	0.95 (0.88–1.02)	0.95 (0.87–1.04)
	Private Health	None	Reference	Reference	Reference
	Insurance	Private	1.01 (0.97–1.05)	0.88 (0.83–0.93)	0.92 (0.86–0.99)
	Work Status	Not working	Reference	Reference	Reference
		Part time	1 (0.94–1.06)	0.93 (0.84–1.04)	0.92 (0.8–1.06)
		Full time	1 (0.91–1.09)	0.87 (0.73–1.03)	0.73 (0.57–0.94)
		Retired due to ill health	1.11 (1.05–1.16)	1.25 (1.17–1.34)	1.46 (1.36–1.58)
**Need**	Self-rated	Excellent or Good	Reference	Reference	Reference
	Health	Poor or very poor	1.11 (1.05–1.17)	1.35 (1.25–1.45)	1.33 (1.21–1.45)
	Self-rated	Excellent or Good	Reference	Reference	Reference
	QOL^2^	Poor or very poor	1 (0.95–1.06)	1.09 (1.01–1.18)	1.18 (1.08–1.28)
	Self-rated memory	Excellent or Good	Reference	Reference	Reference
		Poor or very poor	0.97 (0.93–1.01)	0.89 (0.84–0.95)	0.89 (0.83–0.96)
	# of Chronic Conditions	0 conditions	Reference	Reference	Reference
		1 condition	0.99 (0.94–1.05)	0.97 (0.88–1.06)	0.94 (0.84–1.06)
		2 conditions	1.03 (0.97–1.09)	0.98 (0.89–1.08)	1.03 (0.92–1.15)
		3 conditions	1.03 (0.97–1.1)	1.08 (0.98–1.19)	1.05 (0.93–1.18)
		4+ conditions	1.06 (0.98–1.13)	1.08 (0.97–1.19)	1.05 (0.93–1.19)
	Physical-function Score^3^	MOSPF 90–100	Reference	Reference	Reference
		MOSPF 60–89	1.23 (1.17–1.29)	1.71 (1.55–1.88)	2.87 (2.47–3.34)
		MOSPF 40–59	1.48 (1.39–1.59)	2.58 (2.31–2.88)	4.98 (4.23–5.86)
		MOSPF 20–39	1.69 (1.57–1.82)	3.07 (2.73–3.45)	7.11 (6.02–8.39)
		MOSPF 0–19	1.69 (1.55–1.84)	3.09 (2.71–3.51)	7.37 (6.19–8.78)
	Help with disability	No	Reference	Reference	Reference
		Yes	1.01 (0.95–1.08)	1.16 (1.08–1.26)	1.46 (1.35–1.59)
	Osteoarthritis Treatment^4^	No	Reference	Reference	Reference
		Yes	1.23 (1.18–1.3)	1.56 (1.47–1.66)	1.95 (1.82–2.09)
	Recent^5^ hip replacement surgery	No	Reference	Reference	Reference
		Yes	1.23 (1.01–1.49)	1.3 (1.02–1.68)	1.38 (1.07–1.78)
	Recent^5^ knee replacement surgery	No	Reference	Reference	Reference
		Yes	1.44 (1.26–1.64)	1.57 (1.32–1.87)	1.35 (1.08–1.69)
	Used paracetamol^4^	No	Reference	Reference	Reference
		Yes	1.29 (1.24–1.34)	1.8 (1.7–1.9)	1.7 (1.59–1.82)
	Anxiety treatment^4^	No	Reference	Reference	Reference
		Yes	0.96 (0.88–1.05)	1.03 (0.93–1.15)	0.93 (0.83–1.04)
	Depression Treatment^4^	No	Reference	Reference	Reference
		Yes	1.04 (0.97–1.12)	1.13 (1.03–1.24)	1.2 (1.09–1.32)
	K10 Psychological	Low	Reference	Reference	Reference
	Distress	Moderate	1.03 (0.98–1.08)	1.12 (1.04–1.21)	1.12 (1.03–1.22)
		High	1.07 (0.99–1.16)	1.15 (1.05–1.27)	1.12 (1.01–1.24)
		Very high	1.07 (0.96–1.18)	1.16 (1.02–1.31)	1.08 (0.95–1.23)

1 Language other than English.

2 Quality of Life.

3 Medical-outcomes study physical function scale, values range from 0 (lowest physical function level) to 100 (highest physical function level.

4 Self-reported, in last 28 days.

5 Self-reported surgery within 2 years of baseline recruitment.

## Discussion

The use of long-term opioid analgesic therapy for persistent non-cancer pain remains controversial, with a lack of evidence of effectiveness, particularly in the context of complex patients, and epidemiological evidence of population harms related to large-scale long-term use. This study provides new insights into understanding the characteristic of people who are more likely to receive long-term opioid analgesic treatment, identifying predisposing, access, and need factors in a heterogeneous population-based sample with reasonably equitable access to healthcare.

Not surprisingly, need factors (notably poorer self-reported physical functioning, poorer psychological status etc.) showed the strongest associations with subsequent opioid analgesic treatment. However, predisposing and access factors were also significantly associated with treatment. Participants with better socio-economic status indicators (higher income and education levels, private health insurance, and full-time work status) were less likely to be on longer-term opioid analgesic treatment, as were older participants and those not speaking English at home. Those with poorer health habits (smoking, obesity and low physical activity levels) were more likely to receive subsequent opioid analgesic treatment. Access factors also reflecting better socio-economic status (higher income and private health insurance) were also associated with a lower likelihood of longer-term opioid analgesic treatment. Despite decline in intensity of opioid analgesic use with age, the overall pattern of age-related prescribing in this cohort is similar to that seen in Australian primary care settings [7].

These findings suggest that longer-term opioid analgesic dispensing is being seen in groups who could be seen at higher risk of poor health based on a wide range of health and non-health factors characterised by poor physical and psychological health, and social disadvantage. These findings can be contextualised against known gaps in the evidence for rational long-term opioid analgesic prescribing for non-cancer pain [22]. One of these gaps, addressed by the current study, is the influence of social context on the treatment benefit of opioid pharmacotherapy. The picture emerging from the present study is of complex needs in community-dwelling individuals who are started on longer-term opioid therapy. The complex health needs found in our study are consistent with those found in other cohort studies [23], [24]. Despite the relative equity of access to care in Australia afforded by universal access to publically-subsidised primary care, hospital care and pharmaceuticals, what has also emerged is evidence of another form of ‘adverse selection’, related to non-health factors.

Another novel feature of our study was the use of a prescriber diversity index to assess continuity of prescribing in the opioid treatment groups, as continuity of care is important in long-term management of pain. Longer-term treatment was associated with an increased number of prescribers: while the reasons for this are unclear it does warrant further investigation. This also indicates that the number and distribution of opioid analgesic dispensings between prescribers is similar between these long-term and episodic users.

No inferences about the indications for, or effectiveness of, opioid analgesic prescribing are appropriate, as this was not an aim of the study. It is worth noting that access to specialist pain management services was very limited in NSW at the time of this study [25]. There were strong associations between longer-term opioid analgesic use and a number of indirect indicators of pain status (recent treatment for osteoarthritis, recent joint replacement surgery, recent use of paracetamol) that are broadly in line with Australian primary care data [7]. As well as being an indirect indicator of pain status, recent surgery has been identified elsewhere as a risk factor for long-term opioid use [26].

Strengths of this study include a large sample size that is representative of the community dwelling population, use of a heterogeneous cohort who were not selected on the basis of seeking healthcare, novel methods for defining categories of treatment and for measuring prescriber diversity, and the use of a conceptual framework for studying opioid prescribing at a population level. To our knowledge, this is the first time the Andersen-Newman model has been used in a population-based study of long-term opioid prescribing.

This study has some limitations. Participants in this study may not be fully representative of the Australian community. The *45 and Up Study* was not designed to be representative of the NSW population, with intentional oversampling of those aged ≥80 years and in rural areas [10]. Compared to other studies of people dispensed opioid analgesics, the population sample is likely older, more heterogeneous, and covered by universal health insurance. There may also be a ‘healthy cohort’ effect whereby individuals who participate in studies such as *45 and Up Study* are likely to be in better health than the general community. However, it is important to note that these issues of representativeness do not affect the generalisability of relationships observed within the cohort. Comparison of data from the study with representative studies such as the NSW Population Health Survey (response rate of 70%), has shown that generalisability of the *45 and Up Study* findings are high. Outcome-exposure relationships (e.g. diabetes and Body Mass Index), were very similar between *45 and Up Study* participants and those of the NSW Population Health Survey despite the differences in response rates and representativeness [27]. Our study excluded general beneficiaries due to gaps in data capture; however this likely constituted a small proportion of people dispensed opioid analgesics. More than 85% of all PBS medicines are dispensed to social security beneficiaries in Australia [28] as these individuals are older and in poorer health than the general community [14].

Clinical information is not captured by the PBS, so we did not have diagnosis or pain intensity data for people dispensed opioid analgesics. It is therefore difficult to determine the net impact of people's underlying condition and opioid analgesic treatment on their physical functioning and mental health. Our data were limited to use of PBS-subsidised prescription medicines, and we did not have access to information about non-prescription medicine use or outside of PBS subsidy (‘private’ prescriptions). In particular, we have likely underestimated use of low-dose codeine preparations and laxatives, which are available over-the-counter in Australia.

In conclusion, the boundaries of ‘adverse selection’ for opioid analgesic therapy are wide, even in a population setting where access to healthcare is relatively equitable, and include a complex array of factors not limited to the physical domain. These need to be fully captured and accounted for in evaluating the role of opioid analgesic therapy within populations.

## Supporting Information

Table S1 Sensitivity of opioid analgesic treatment group algorithm with additional PBS service records (+6 months retrospective data).
(DOCX)
