# Supplementary material for: Adverse Selection? A Multi-Dimensional Profile of People Dispensed Opioid Analgesics for Persistent Non-Cancer Pain
Source: PLoS One. 2013 Dec 2;8(12):e80095. doi: 10.1371/journal.pone.0080095 (PMC3846564; doi:10.1371/journal.pone.0080095)
Supplement: Table S1 — Sensitivity of opioid analgesic treatment group algorithm with additional PBS service records (+6 months retrospective data). (DOCX) [file pone.0080095.s001.docx]

| **1 year prospective** | **1 year prospective + 6 months retrospective** | | | |  |
| --- | --- | --- | --- | --- | --- |
| **period** | No Opioid | Acute | Episodic | Long-term | Total |
| No Opioid | 73120 | 4059 | 320 | 46 | 77545 |
| Acute | - | 8892 | 1751 | 135 | 10778 |
| Episodic | - | - | 4653 | 206 | 4859 |
| Long-term | - | - | - | 3505 | 3505 |
